# A Case of Congenital Lymphedema Complicated by Chronic Chylous Effusions and Recurrent Pericardial Effusion Requiring Pericardial Window

**DOI:** 10.7759/cureus.8160

**Published:** 2020-05-16

**Authors:** Abdulrahman Katabi, Mohammed Al-Ourani, Fuad Zeid

**Affiliations:** 1 Internal Medicine, Joan C. Edwards School of Medicine, Marshall University, Huntington, USA; 2 Pulmonology and Critical Care, Joan C. Edwards School of Medicine, Marshall University, Huntington, USA; 3 Pulmonary Medicine, Joan C. Edwards School of Medicine, Marshall University, Huntington, USA

**Keywords:** chylothorax, chylopericardium, pleural effusion, primary lymphedema, cardiac tamponade

## Abstract

We are presenting a case of primary lymphedema (PL) complicated with a repeated need for thoracentesis and pericardiocentesis. Our patient is a 24-year-old male with primary lymphedema that is manifested in the left hand and right lower limb. The patient presented to the emergency department (ED) for recurrent right lower lobe cellulitis that had failed repeated attempts with outpatient antibiotic therapy. The patient was admitted to the intensive care unit due to signs of cardiac tamponade that were discovered on the physical examination. Pericardial tamponade was confirmed by echocardiography. The patient underwent thoracentesis and multiple pericardiocenteses and required a pericardial window. Pericardial and pleural fluids appeared milky and biochemical analysis was consistent with chylopericardium and chylothorax.

## Introduction

Chylothorax is a lymphatic fluid collection that occurs in the pleural space. The fluid collection results from interruption of the lymphatic system circulation in the thorax. This may be due to primary lymphatic disease or secondary to trauma, tumors, or chest surgeries. Primary lymphedema (PL) presents with systemic symptoms and fluid collections that may include spontaneous chylothorax, chylopericardium, limb lymphedema, scrotal lymphedema, and ascites [1].

## Case presentation

A 24-year-old male with a history of congenital lymphedema affecting both the left upper extremity and right lower extremity was admitted for recurrent cellulitis of the right leg that failed outpatient therapy. In addition to obstructive sleep apnea and asthma, he had a history of lymphocytic transudative pericardial effusion requiring pericardiocentesis a year ago. Home medications included Advair, Ventolin, and Lasix.

Physical examination

**Figure 1:**
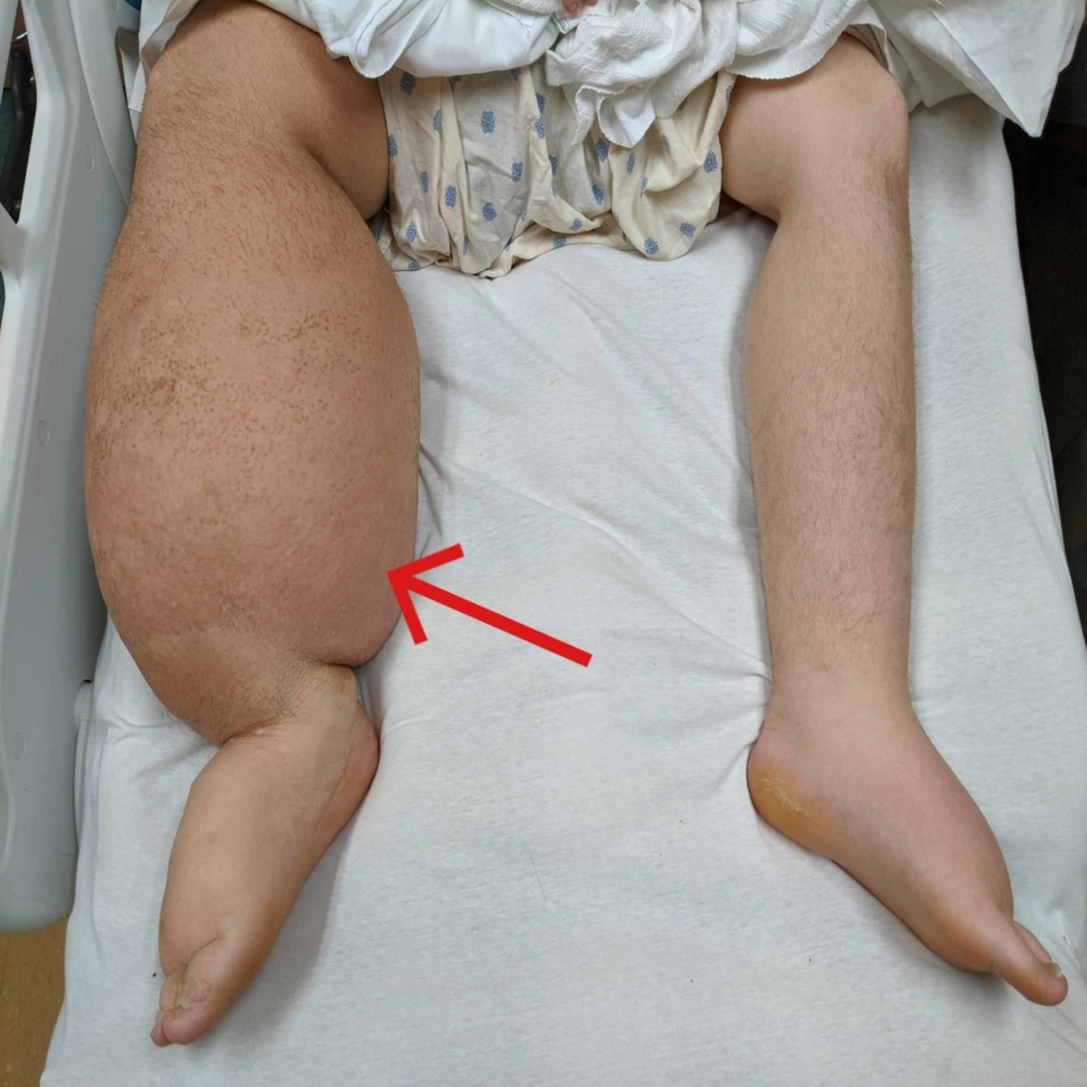
Right leg lymphedema

**Figure 2:**
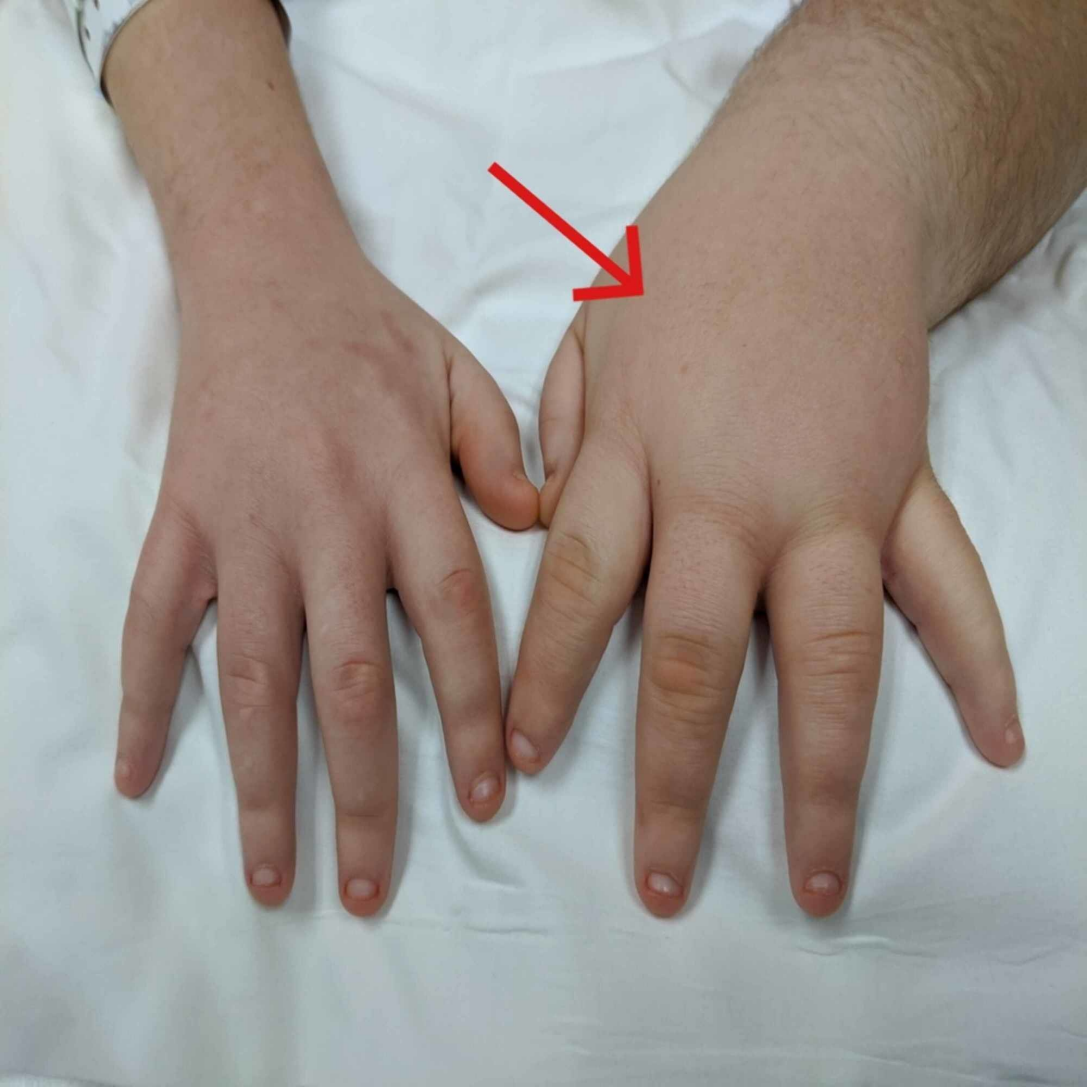
Left hand lymphedema

Vital signs showed temperature 98.4 F, blood pressure 124/61 mmHg, pulse 107 bpm, arterial oxygen saturation 100% on room air, and respiratory rate 18/min. No pulsus paradoxus was noted on the exam. The patient is obese, with right lower extremity edema with redness and increased warmth of the skin and left upper extremity edema (Figures 1, 2). Heart sounds were muffled. Neck veins were distended. Breath sounds were diminished over the lung bases. Other physical exam findings showed scrotal edema.

Diagnostic and therapeutic procedures

**Figure 3:**
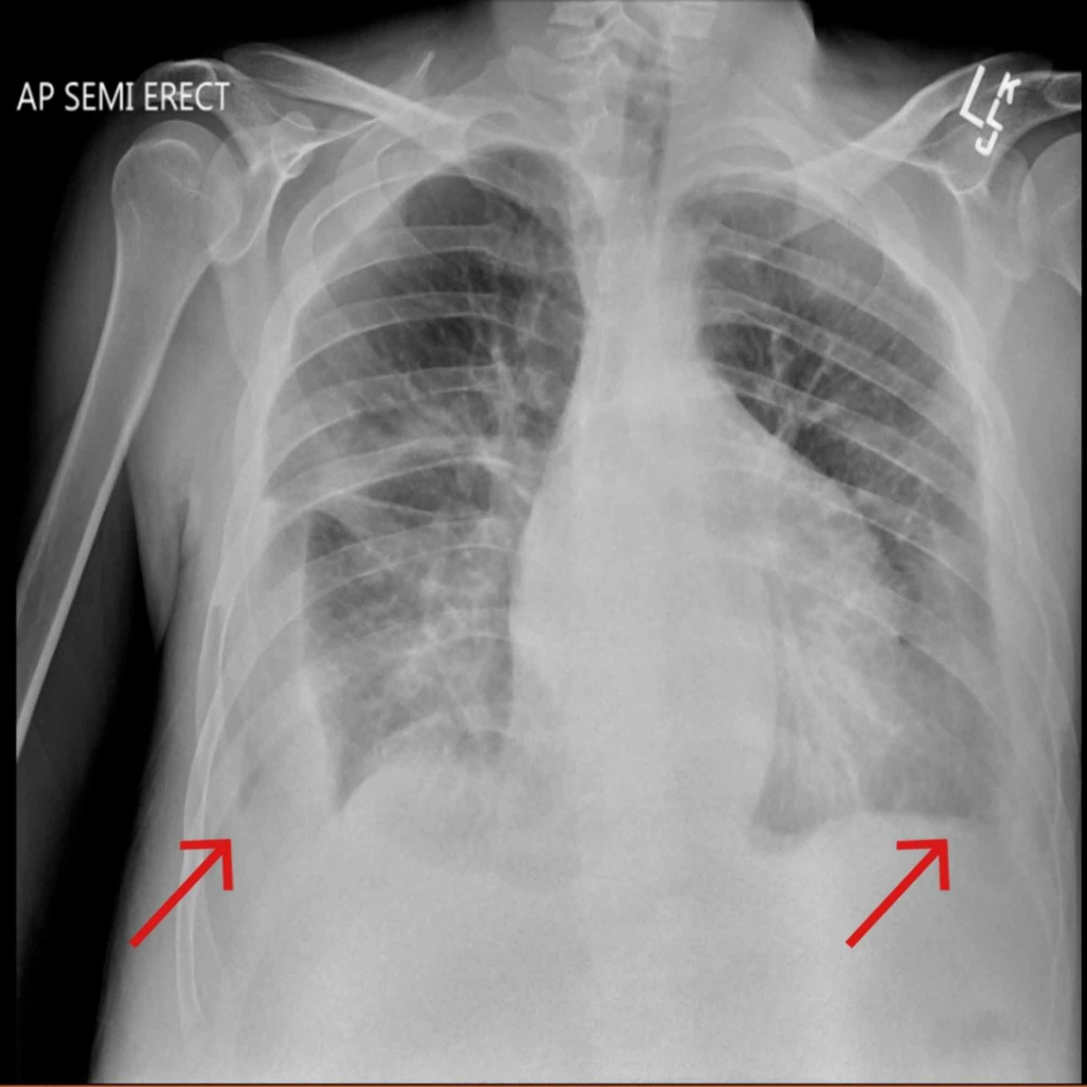
Initial chest X-ray - the arrows are showing the pleural effusions

**Figure 4:**
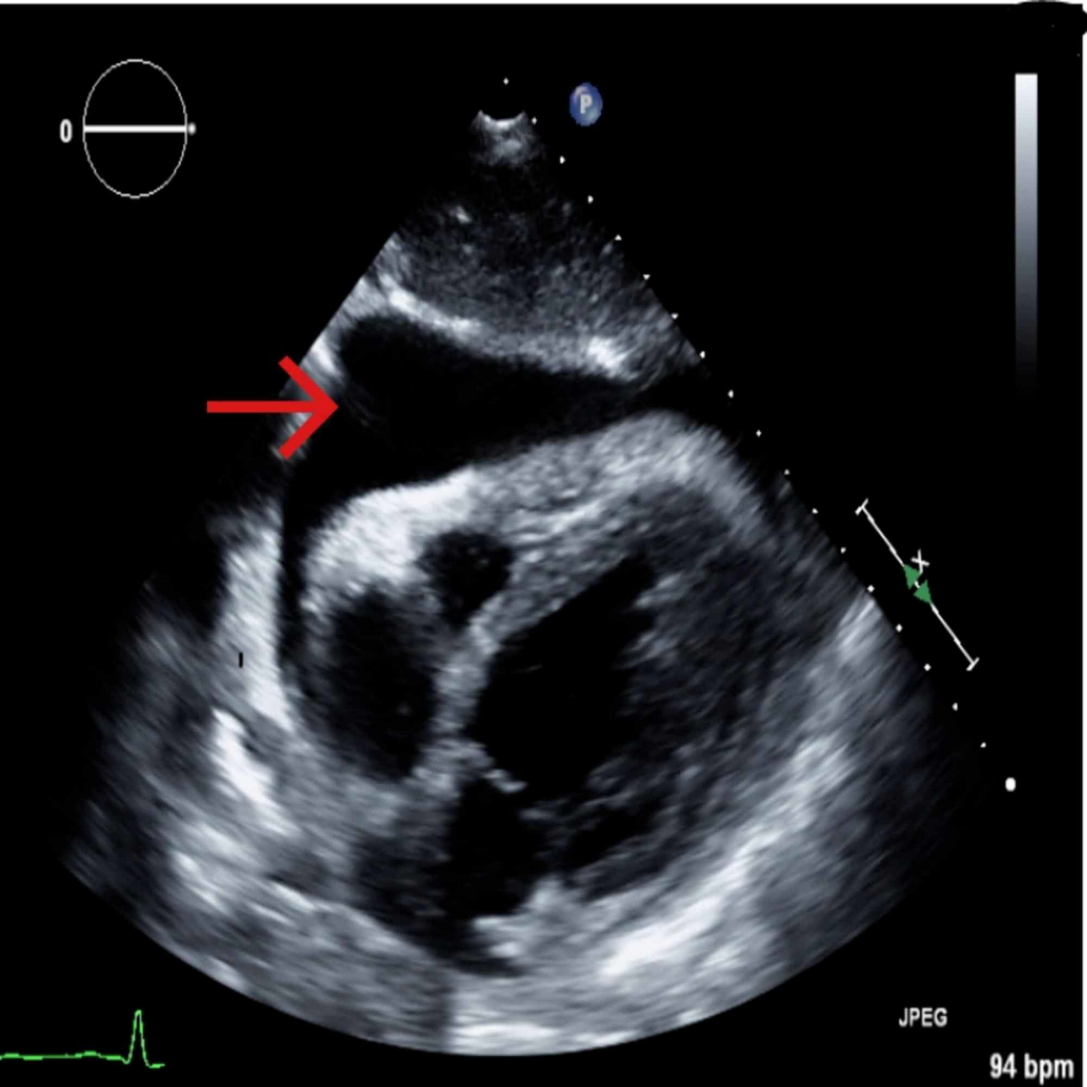
Pericardial effusion as indicated by the arrow

**Figure 5:**
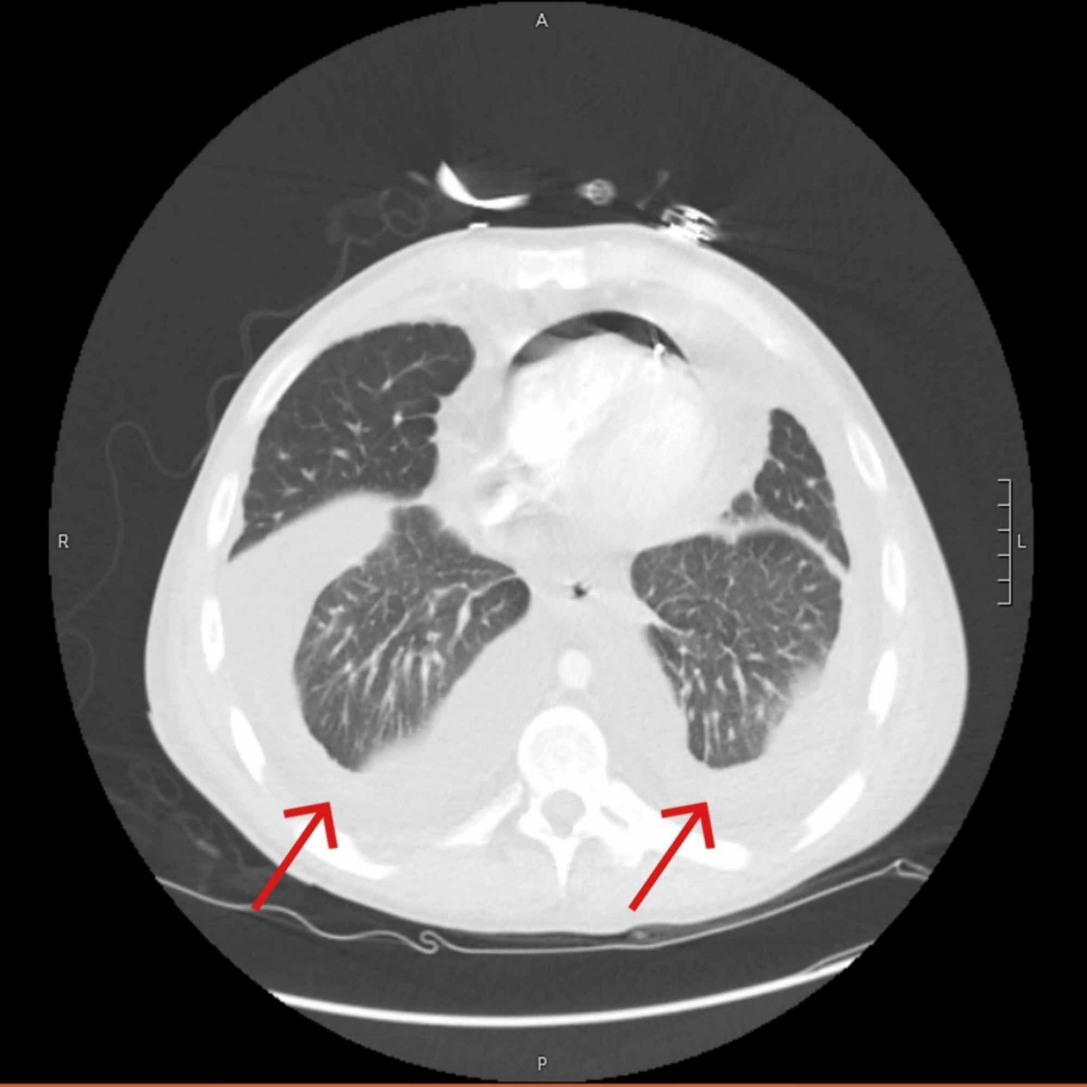
Chest CT showing bilateral pleural effusions

**Figure 6:**
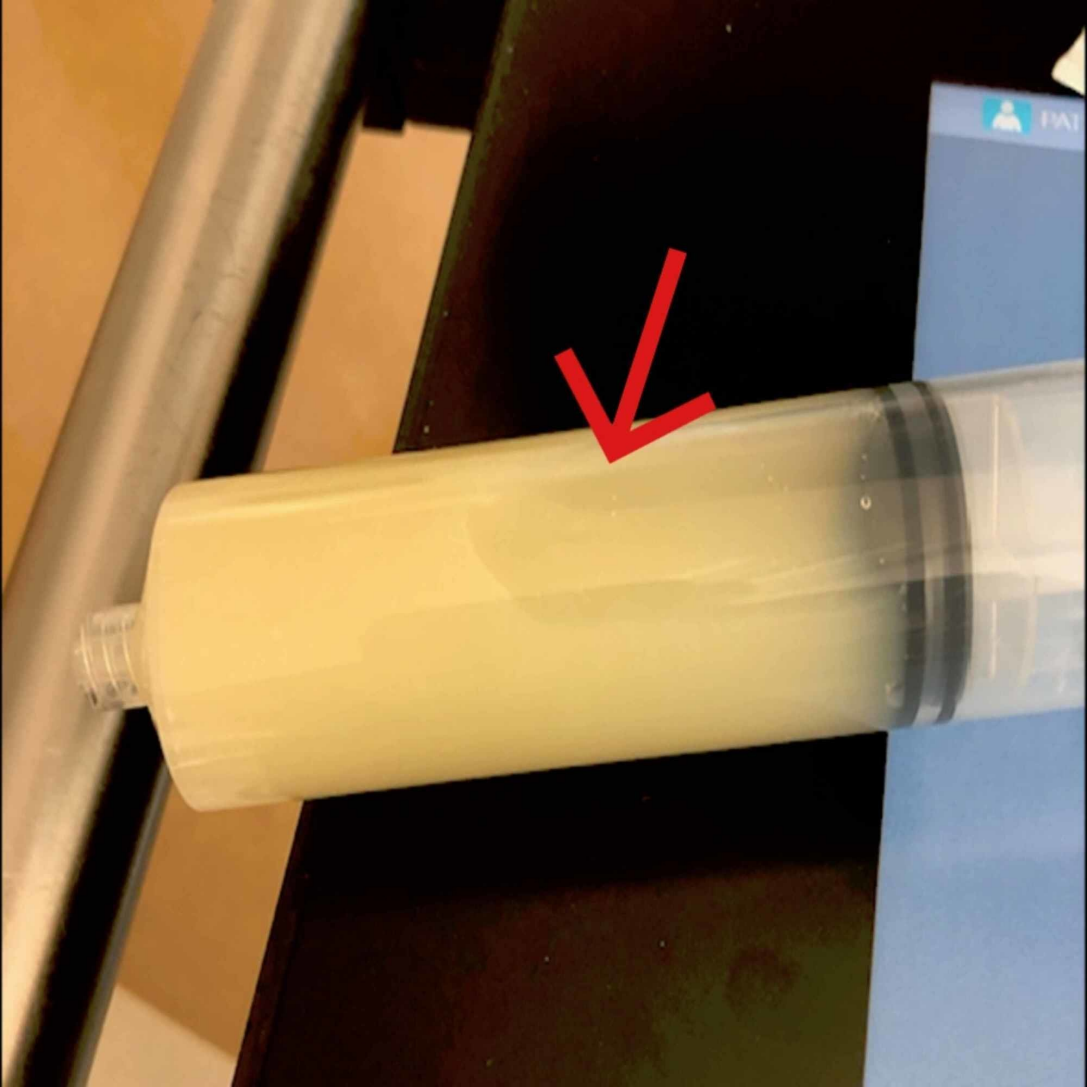
Chylous fluid from thoracentesis

Complete blood count showed WBCs at 11.3 K/cmm. Lab test showed albumin 1.9, total protein 4.9, and B-type natriuretic peptide 383 pg/mL. Chest radiograph (Figure 3) showed mild edema with bilateral pleural effusions and pericardial effusion. Echocardiogram (Figure 4) showed moderate to large pericardial effusion mostly anterior to the right ventricle with early tamponade and excessive respiratory variation present. Pericardiocentesis drained 380 ml of straw-colored fluid. Pericardial fluid analysis showed WBCs 95, RBCs 3273, neutrophils 42%, lymphocytes 47%, albumin 1.5, cholesterol < 50, glucose 100, LDH 145, PH 7.86, protein 2.4, triglyceride 7. Chest CT with IV contrast after the pericardiocentesis, showed no residual pericardial effusion but also showed bilateral pleural effusions more on the right side (Figure 5). Ultrasound-guided thoracentesis obtained 500 ml of milky appearing fluid (Figure 6). Pleural fluid analysis showed WBCs 77, RBCs 37, neutrophils 1%, lymphocytes 65%, albumin 1.1, amylase 15, cholesterol < 50, LDH 35, PH 7.0, protein 1.9, TG 284. Cultures and cytology studies were negative. The patient required pericardial window for control of recurrent pericardial effusion later on.

## Discussion

The disease mechanism of the PL depends on the imbalance between capillary dynamics and movement of the interstitial fluid due to lymphatic anomalies. When this mechanism is interrupted or impaired, the lymphatic fluid accumulates in the interstitial space and causes reactive fibrosis of the surrounding tissues.

PL is a hereditary disease. Types of PL are categorized according to the age at presentation: 1) at birth, called congenital hereditary lymphedema or Milroy disease; 2) at puberty, called lymphedema praecox; 3) during the third decade of life, called hereditary lymphedema tarda. Turner’s syndrome and Noonan’s syndrome are also associated with PL. On the other hand, secondary lymphedema is usually due to interruption of the lymphatic system drainage which could happen with malignancy, infection, trauma, or surgery [2]. Primary lymphedema occurs in 1:6,000 to 1:10,000 people. It is a rare condition and the treatment is challenging due to recurrence [3-6]. Treatment goal in chylothorax is to decrease the flow through the thoracic duct and it can be achieved either surgically or medically. Choosing the management strategy depends on the amount of the chylothorax per day, if >1 L / day then it needs surgical or interventional solutions. Low output chylothorax can be managed by dietary modifications with or without medications. Dietary changes include a high protein diet with low fat intake and replacement of fat-soluble vitamins [7]. Somatostatins, octreotide and midodrine can decrease the flow of the chyle in the thoracic duct by several mechanisms. Octreotide and somatostatin decrease the chyle volume by decreasing the absorption of the triglycerides from the intestine. Midodrine causes vasoconstriction to the lymphatic vessels [8,9].

On reviewing the literature about the chemical classification of pericardial fluid, we found that there is an obvious debate about the clinical usefulness of using Light's criteria in classifying the pericardial fluid. In Ben-Horin et al. study, they mentioned that there is no clinical applicability of using Light's criteria in pericardial disease as most of their patients in the study had exudative results (118 of 120). In Burgess et al. study, the majority of their patients had exudative pericardial fluid as well (94 of 112), but they concluded that Light's criteria is a reliable tool and it has high sensitivity in detecting exudative pericardial effusions (98%). In another study, Cale-Subia and De Luna took the middle position in the debate and concluded that we still can use Light's criteria in pericardial effusion but with caution due to higher lactate dehydrogenase (LDH) and protein levels in the pericardial fluid compared to the pleural fluid [10-12]. The treatment of chylopericardium is mostly conservative at the beginning, but the failure percentage of the conservative approach is high at 57.1% and these patients end up needing more invasive management like a pericardial window or surgery [13].

## Conclusions

More data are needed on clinical benefits of screening for pleural and pericardial effusions in primary lymphedema (PL) patients regardless of symptoms. Because PL is rare and chylothorax in the setting of PL is even rarer, there is limited evidence available to guide management.
